# Integrated Analysis of Long Noncoding RNA and Coding RNA Expression in Esophageal Squamous Cell Carcinoma

**DOI:** 10.1155/2013/480534

**Published:** 2013-10-07

**Authors:** Wei Cao, Wei Wu, Fachun Shi, Xiaobing Chen, Lihua Wu, Ke Yang, Fu Tian, Minghui Zhu, Guoyong Chen, WeiWei Wang, Fred G. Biddle, Jianqin Gu

**Affiliations:** ^1^Clinical Research Center, People's Hospital of Zhengzhou, 33 Yellow River Road, Zhengzhou, Henan 45003, China; ^2^Department of Pathology and Experimental Medicine, University of Calgary, Calgary, AB, Canada T2N 4N1; ^3^Science and Education Department, Health Bureau of Zhengzhou, China; ^4^Departments of Medical Genetics and Biological Sciences, University of Calgary, Calgary, AB, Canada T2N 4N1

## Abstract

Tumorigenesis is a complex dynamic biological process that includes multiple steps of genetic and epigenetic alterations, aberrant expression of noncoding RNA, and changes in the expression profiles of coding genes. We call the collection of those perturbations in genome space the “cancer initiatome.” Long noncoding RNAs (lncRNAs) are pervasively transcribed in the genome and they have key regulatory functions in chromatin remodeling and gene expression. Spatiotemporal variation in the expression of lncRNAs has been observed in development and disease states, including cancer. A few dysregulated lncRNAs have been studied in cancers, but the role of lncRNAs in the cancer initiatome remains largely unknown, especially in esophageal squamous cell carcinoma (ESCC). We conducted a genome-wide screen of the expression of lncRNAs and coding RNAs from ESCC and matched adjacent nonneoplastic normal tissues. We identified differentially expressed lncRNAs and coding RNAs in ESCC relative to their matched normal tissue counterparts and validated the result using polymerase chain reaction analysis. Furthermore, we identified differentially expressed lncRNAs that are co-located and co-expressed with differentially expressed coding RNAs in ESCC and the results point to a potential interaction between lncRNAs and neighboring coding genes that affect ether lipid metabolism, and the interaction may contribute to the development of ESCC. These data provide compelling evidence for a potential novel genomic biomarker of esophageal squamous cell cancer.

## 1. Introduction

Esophageal squamous cell carcinoma (ESCC) is one of the most common types of cancer, and it ranks among the main causes of cancer deaths worldwide [1]. There are marked regional variation and exceptionally high incidence in certain areas of China. Despite advances in multidisciplinary treatment of ESCC, 5-year survival rate remains poor. The initiatome [2] of ESCC is a complex dynamic biological process in genome space, and it may include multiple steps of genetic and epigenetic alterations [3], aberrations in expression of noncoding RNA (e.g., microRNAs) [4], and changes in the expression profile of coding genes [5, 6]. In past decades, expression profiling of coding genes has defined important signaling pathways involved in tumorigenesis. The latest knowledge of actively transcribed long noncoding RNAs (lncRNAs) from high-throughput sequencing is revealing an even greater complexity about cancer genome regulatory networks.

LncRNAs are endogenous cellular RNA transcripts, ranging from 200 to 100,000 nucleotides in length, and they lack an open reading frame of significant length (less than 100 amino acids) [7]. LncRNAs are generally expressed at a lower level than protein-coding genes, and they display more tissue-specific and cell-specific expression patterns [8, 9]. LncRNAs were previously believed to be transcriptional noise, but now they have critical roles in development and differentiation as well as in the proliferation and progress of disease, including cancer [10]. Mechanisms of action of transcribed lncRNAs are described as modifying chromatin architecture and regulating gene expression in a cis or trans manner. For example, H19 lncRNA cis-regulates *IGF2* gene expression at the same genomic locus, and HOTAIR lncRNA is transcribed on Chr 12, and it transregulates *HoxD* gene on Chr 2. Additionally, lncRNAs have also been reported to coordinate the regulation of neighboring coding genes through a “locus control” process [11], which mediates the localization of genes within nuclear regions to favor their transcription through the formation of domains of histone modification and intra- or interchromosomal loops [12]. Dysregulated lncRNAs have been identified with different screening methodologies in various types of cancer. For example, the cancer-related lncRNA, metastasis-associated lung adenocarcinoma transcript 1 (*MALAT-1*), was identified by subtractive hybridization during screening for early non-small cell lung cancer with metastasis [13]. Overexpression of *MALAT-1* is highly predictive of poor prognosis and shortened survival time in early stage lung cancer. Overexpression of *HOTAIR* lncRNA was found in several solid tumors [14–17] in association with cancer metastasis, and increased *HOTAIR* expression in breast cancer is transcriptionally induced by estradiol [18]. Prostate cancer associated lncRNA, PCGEM1 [19], and *PCAT-1* [20] appear to be prostate-specific regulators of cell apoptosis and proliferation. Recently, *AFAP1-AS1* lncRNA was reported to be overexpressed in esophageal adenocarcinoma [21]. 

The handful of dysregulated lncRNAs in different cancers suggests that lncRNAs are an enigmatic component of the whole transcriptome, which may participate in tumorigenesis, invasion, and metastasis. Efforts are being made to explore the “lncRNAome” of various cancers with advanced high-throughput RNA sequencing technologies [8, 9], and dynamic changes in lncRNA expression have been observed in cancer cells during different stages of cancer development and during treatment [22]. However, our understanding of the role of lncRNAs in cancer biology is still in an early stage, and a clearly defined, predictive set of biological functions for lncRNAs is lacking in cancer biology. Therefore, thorough searches and analyses of the interactions between lncRNA and coding genes may help to infer their potential biological roles.

In order to understand the role of lncRNAs in ESCC, we report a pilot study of the profiles of differentially expressed lncRNAs and coding RNAs from tumor and adjacent normal tissue of individual patients with ESCC. We assessed the whole transcriptomic landscape for potential interactions between lncRNAs and coding-gene expression. In particular, we evaluated the coding genes that are co-located and co-expressed with the differentially expressed lncRNAs during the genesis of ESCC.

## 2. Results and Discussion

### 2.1. Transcriptomic Landscape of ESCC

**Figure 1:**
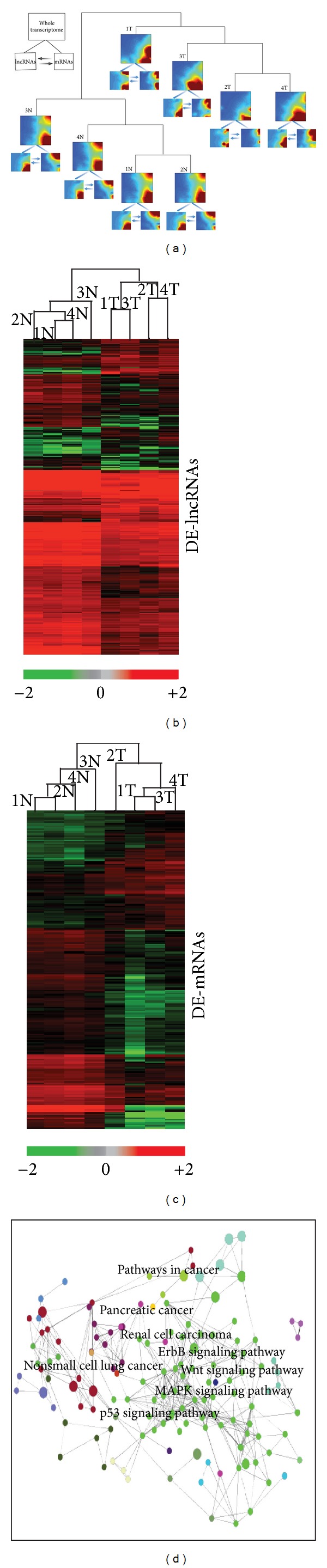
Transcriptomic landscape of esophageal squamous cell cancer (ESCC). (a) Whole transcriptome of tumor (T) and adjacent normal tissue (N) of four patients with ESCC were detected using a microarray with 7,419 long noncoding RNAs (lncRNAs) and 27,958 coding RNAs. Two main clusters (Ts and Ns) were generated using unsupervised clustering methods. Then, a self-organizing map (SOM) of either whole transcriptome (both lncRNAs and mRNAs) or lncRNAs or mRNA was produced from each sample (see legend in up-left corner of this figure, and the arrows are meant to indicate the potential interaction), using gene expression dynamic inspector (GEDI). Mosaic patterns are pseudocolored SOMs to show integrated biological entity in each sample. Red through blue color indicates high to low expression level. (b) and (c) Differentially expressed lncRNAs (DE-lncRNAs) and coding RNAs (DE-mRNAs) in ESCC. Hierarchical clustering analysis of 410 DE-lncRNAs (b) and 1219 DE-mRNAs (c) between ESCC tissue and adjacent normal tissue (fold change > or < 2-fold and *P* < 0.05). Red and green colors indicate high and low expression, respectively. In the heatmap, columns represent samples, and rows represent each gene. The scale of expression level is shown on the horizontal bar. (d) KEGG functional analysis of DE-mRNA networks in ESCC. The DE-mRNA genes are involved in cancer-related signaling functions, and a detailed list of significant GO terms is shown in Figure S1 and its associated legend in Supplementary Information.

Our genome-wide gene expression profiling of both lncRNAs and coding genes from ESCC and adjacent nonneoplastic tissue was conducted to detect possible associations of lncRNAs with ESCC. We first asked whether these transcripts of 7,419 noncoding and 27,958 coding RNAs could distinguish ESCC from normal tissues.  Figure 1(a) shows that the four ESCC samples are clustered together in one group and clearly separated from the samples of normal tissue. Next, we examined the whole transcriptomic pattern (lncRNAs + coding RNAs) from each sample and the landscapes of the whole transcriptome (represented by heatmaps in Figure 1(a)) of normal tissues differ from those of ESCC that exhibit more heterogeneous alterations. The overall changes from a respective normal to cancer state were also seen separately as a difference in expression profile of either the lncRNA or the coding RNA. These observations suggest that a potential dynamic interaction between lncRNAs and coding RNAs may be reshaping the landscape of the whole transcriptome during ESCC development. 

To gain a detailed understanding of the biological themes of all RNA transcripts, we further identified those transcripts that are significantly and differentially expressed (DE) in ESCC tissue compared to matched normal tissue, based on the criteria described in the methods. There are 410 DE-lncRNAs and 1219 DE-mRNAs that represent about 5% of the transcripts in the respective microarrays (Supplementary Tables S1 and S2 available online at http://dx.doi.org/10.1155/2013/480534). DE-lncRAs distinguish a cancer cell from its normal cell state with three times fewer transcripts than DE-mRNAs (Figures 1(b) and 1(c)), suggesting that the DE-lncRNA profile is more informative and, potentially, a more faithful indicator of a specific cell state. 

Enrichment analysis of DE-mRNAs demonstrated that the respective genes are involved in cancer-related pathways (Figure 1(d)). Since expression profiling of coding-RNA has been intensively studied in esophageal cancer, we validated 10 genes whose expression level in other studies [23–25] is significantly changed (*P* < 0.05) by at least 2-fold relative to normal tissues (Table 1). 

### 2.2. Expression of lncRNAs in ESCC

**Figure 2:**
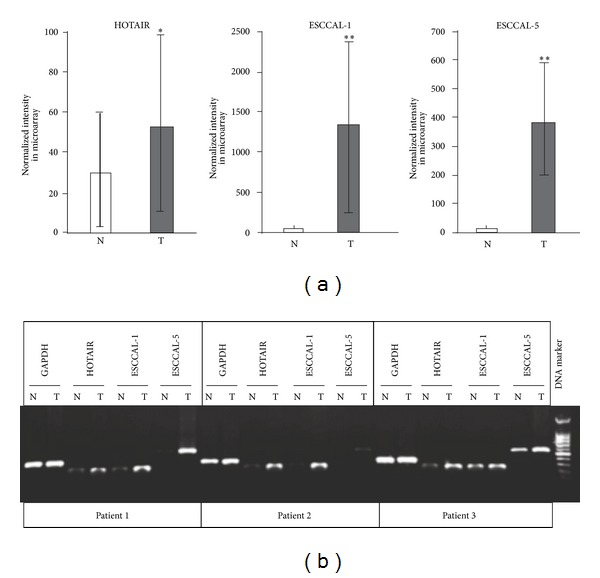
Long noncoding RNAs (lncRNAs) expression in esophageal squamous cell carcinoma (ESCC). (a) Three differentially expressed lncRNAs, HOTAIR, ESCCAL-1, and ESCCAL-5, from microarray detection. The average intensity of expression in normal tissues (N) and tumors (T) is plotted with their standard deviations. (b) Validation of HOTAIR, ESCCAL-1, and ESCCAL-5 with independent patient samples by PCR analysis. The amplicons were separated with 2% agarose gel. GAPDH was used as an internal control. Significance is *P < 0.05, **P < 0.01.

LncRNAs are emerging as a novel class of noncoding RNAs that are pervasively transcribed in the genome, but there is limited functional knowledge about them. High-throughput screening of lncRNAs from ESCC has been poorly studied, except for a recent report of overexpressed lncRNA, AFAP1-AS1, in esophageal adenocarcinoma [21]. In our study, a total of 7,419 intergenic lncRNAs and other transcripts of uncertain coding potential were examined, and we identified 410 DE-lncRNAs in ESCC relative to adjacent normal esophageal tissues. We named the anonymous lncRNAs ESCC Associated Long noncoding RNAs (ESCCAL, Supplementary Table S1). Expression of HOTAIR lncRNA is increased in various cancers [14–17, 26], and it is also significantly increased in our analysis of ESCC (Figure 2(a)). In addition, we confirmed another two upregulated lncRNAs that are differentially expressed in ESCC and that we have named ESCCAL-1, and ESCCAL-5. The increased and differential expression of HOTAIR, ESCCAL-1 and ESCCAL-5 in ESCC tissue relative to adjacent nonneoplastic tissue was independently assessed with PCR methods in matched-pair tissue samples from three additional ESCC patients and the results are consistent with the microarray analysis (Figure 2(b)). Interestingly, except for HOTAIR, other previously reported lncRNAs (i.e., MALAT-1, PCAT-1 and AFAP1-AS1) are not differentially expressed in our analysis of ESCC. Therefore, the DE-lncRNAs that we have identified may be a unique property of ESCC, and we are currently using a population-based analysis to characterize these DE-lncRNAs as potential genomic biomarkers and regulatory elements in the dynamic process leading to ESCC. 

### 2.3. LncRNAs Co-located and Co-expressed with Coding Genes in ESCC

**Figure 3:**
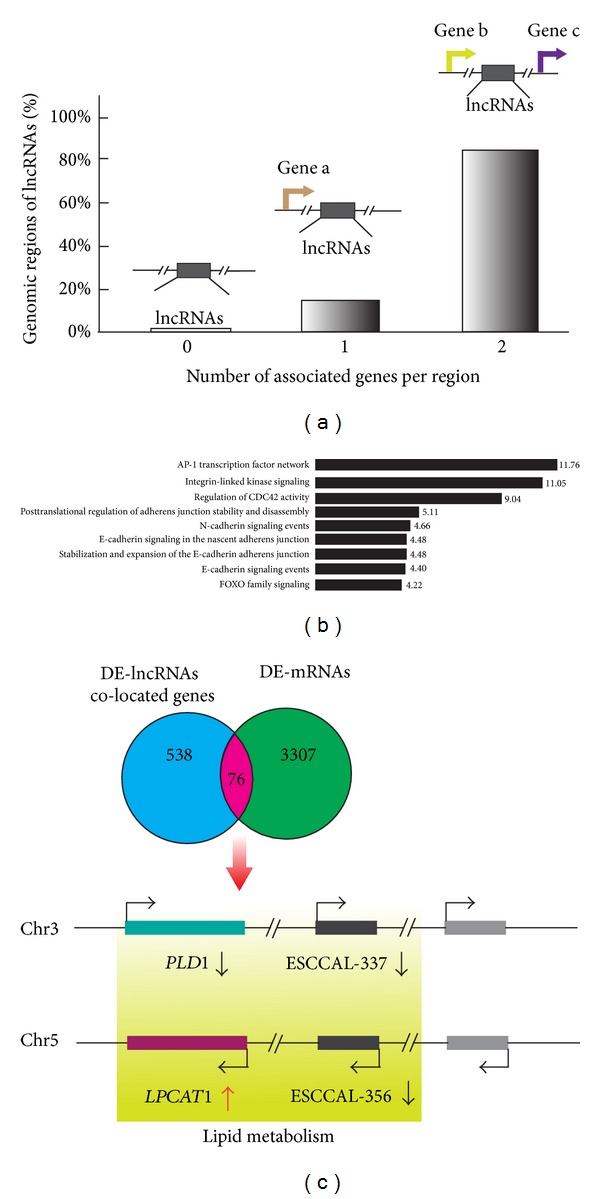
Identification of lncRNAs co-located and co-expressed neighboring genes in esophageal squamous cell carcinoma (ESCC). (a) Identification of neighboring genes of the DE-lncRNAs. The genomic coordinate information of 410 DE-lncRNAs was used to search neighboring genes whose genomic locations are within ~5 kb upstream and ~1 kb downstream of the lncRNA and may extend to 1000 kb in both directions using GREAT software (http://bejerano.stanford.edu/great/public/html/index.php). The percentage of DE-lncRNAs harboring zero, one, or two neighboring genes is presented. (b) Gene Ontology (GO) enrichment analysis of lncRNAs co-located genes. Identified gene enriched pathways/terms are listed on the left; the length of horizontal bars and the numbers on the right indicate the percentage of genes involved in each pathway/term. (c) LncRNAs co-located and co-expressed coding mRNAs. Overlap of 538 DE-lncRNA co-located genes with 3307 DE-mRNAs in microarrays identified 76 lncRNAs co-located and co-expressed coding mRNAs (list in Table 2). GO enrichment analysis suggests phospholipase D1 (*PLD1*) and lysophosphatidylcholine acyltransferase1 (*LPCAT1*) are involved in ether lipid metabolism pathway. Genomic location shows that *PLD1* is located at −22,068 bp upstream of ESCCAL-337 lncRNA on Chr 3 and *LPCAT1* is at −21,250 bp upstream of ESCCAL-356 lncRNA on Chr 5.

LncRNAs have been reported to coordinate the regulation of neighboring coding genes through a “locus control” process [11]. We wondered whether such a “locus control” process could operate in ESCC development, and, therefore, we searched neighboring genes of the 410 DE-lncRNAs in the genome. The majority (98.8%) of the 410 DE-lncRNAs harbor neighboring coding genes whose genomic locations are within ~5 kb upstream and ~1 kb downstream of the lncRNA and may extend to 1000 kb in both directions (Figure 3(a)). Interrogation of 538 coding genes that are neighbors of these DE-lncRNAs (DE-lncRNAs co-located genes) revealed predicted functions in 9 common pathways such as the AP1 transcription factor network, integrin-linked kinase signaling, several signaling pathways in adherens junctions, and FOXO family signaling (Figure 3(b)).

 We asked whether any DE-lncRNAs co-located genes are also differentially expressed in ESCC. Analysis of the DE-lncRNAs co-located genes with DE-mRNA data set identified 76 genomically co-located and differentially co-expressed genes (Figure 3(c) and Table 2). Strikingly, the co-located and co-expressed genes with DE-lncRNAs may be involved in ether lipid metabolism pathways by the participation of the *LPCAT1* gene encoding lysophosphatidylcholine acyltransferase1 and the *PLD1* gene encoding phospholipase D1 (Figure 3(c)). The lncRNA ESCCAL-337 (chr3:171506370-171528740) was downregulated in ESCC and located at 22,068 bp downstream of the *PLD1* gene, whose expression was also decreased in ESCC. In contrast, the lncRNA *ESCCAL-356* (chr5:1544500-1567142 reverse strand) was downregulated in ESCC and located at 21,250 bp upstream of *LPCAT1*, whose expression was upregulated in ESCC (Figure 3(c)). LPCAT1 modulates phospholipid composition by catalyzing lysophosphatidylcholine into phosphatidylcholine, and overexpression of LPCAT1 was reported to create favorable conditions for cancer cell proliferation [27, 28]. Therefore, at least two of the DE-lncRNAs have the potential to contribute to ESCC by a “locus control” process with neighboring coding genes.

In conclusion, we performed a genome-wide survey of the expression of lncRNAs and coding mRNAs from paired samples of primary neoplastic tissue and adjacent nonneoplastic normal tissue from four individuals. The overall transcriptomic landscape (both lncRNAs and mRNAs) is able to distinguish malignant from normal tissue in each person. We discovered a set of differentially expressed lncRNAs and their co-located and co-expressed coding mRNAs and demonstrated that lncRNAs may be involved in ether lipid metabolism in ESCC. Our study provides genomic support for a model of a “locus control” process in ESCC and a framework for further experimental study. 

## 3. Materials and Methods

### 3.1. Specimens

Written informed consent was obtained from patients before surgery, and the study protocol was approved by the Institutional Review Board for the use of human subjects at Zhengzhou Hospital. Primary tumors and adjacent nonneoplastic tissues were obtained from patients with ESCC who underwent surgical treatment at Linxian Hospital in May 2012. All tissues were frozen in liquid nitrogen immediately after surgical resection. None of the patients had prior chemotherapy or radiotherapy, nor did they have any other serious diseases. All ESCC tissues were histopathologically diagnosed by at least two independent senior pathologists. 

### 3.2. Microarray Hybridization

Total RNAs were extracted using Trizol reagent, following manufacturer's instructions (Invitrogen, Carlsbad, CA, USA). The quality of RNAs was measured with a 2100 Bioanalyzer (Agilent technology, USA). Input of 100 ng of total RNA was used to generate Cyanine-3 labeled cRNA, according to the Agilent One-Color Microarray-Based Gene Expression Analysis Low for Input Quick Amp Labeling kit (v6.0). Samples were hybridized on Agilent SurePrint G3 Human GE 8 × 60 K Microarray (Design ID 028004). Arrays were scanned with the Agilent DNA Microarray Scanner at a 3 *μ*m scan resolution, and data were processed with Agilent Feature Extraction 11.0.1.1. The microarray data discussed in this article have been deposited in National Center for Biotechnology Information (NCBI) Gene Expression Omnibus (GEO) and are accessible through (GEO) Series accession number GSE45350 (http://www.ncbi.nlm.nih.gov/geo/query/acc.cgi?acc=GSE45350).

### 3.3. Validation by Polymerase Chain Reaction (PCR)

 PCR analysis was performed on additional matched ESCC and adjacent nonneoplastic tissues for selected lncRNAs. The primer sequences for PCR are as follows: HOTAIR, forward 5′-GGTAGAAAAAGCAACCACGAAGC-3′ and reverse 5′-ACATAAACCTCTGTCTGTGAGTGCC-3′; ESSCAL-1 (chr8:76121095-76189420 reverse strand), forward 5′-CCAGACAGCAGCAAAGCAAT-3′ and reverse 5′-GGAAGCAGCAAATGTGTCCAT-3′; ESSCAL-5 (chr2:216585154-216585719 forward strand), forward 5′-TACCAACATTGTCCACCGGG-3′ and reverse 5′-GCTGATGACAGTCCCTTGCT-3′. GAPDH was used as a control, forward 5′-CCGGGAAACTGTGGCGTGATGG-3′ and reverse 5′-AGGTGGAGGAGTGGGTGTCGCTGTT-3′. The thermocycle conditions are as follows: initial denaturation at 95°C for 10 minutes, followed by 94°C for 45 seconds, 65°C for 30 seconds, and 72°C for 1 minute for 15 cycles. Then, the annealing temperature was reduced by 0.5°C/cycle for the next 14 cycles, and the amplification was finished with another 24 cycles with the annealing temperature at 58°C. Final extension was at 72°C for 10 minutes. The amplicons were resolved in 2% agarose gel. 

### 3.4. Bioinformatic Analysis

Intensity data were exported to GeneSpring 12.0 (Agilent Technologies, Santa Clara, CA, USA) for quantile normalization and the analysis of differentially expressed long noncoding RNAs and coding RNAs. Paired *t*-test analysis was used to obtain probe sets whose magnitude of change in expression of RNAs between ESCC tissue and adjacent normal esophageal tissue was either greater or less than 2.0 fold and *P* value < 0.05 (*P* values were corrected for multiple testing using the method of Benjamini-Hochberg). The normalized data containing 42544 probes were further analyzed using the R program. All control probes were removed. We then defined the coding (“NM_,” “XM_”) and noncoding (“lincRNA,” “NR_,” and “XR_”) genes in the normalized data according to the definition of RefSeq accession format (http://www.ncbi.nlm.nih.gov/projects/RefSeq/key.html). Differentially expressed long noncoding RNAs (DE-lncRNAs) and coding RNAs (DE-mRNAs) were further identified. The landscapes of the whole transcriptome (lncRNAs + coding RNAs) or all lncRNAs or all coding RNAs were analyzed with gene expression dynamic inspector (GEDI). 

### 3.5. Co-Location and Co-Expression Analysis between DE-lncRNAs and DE-mRNAs

Genomic coordinates of DE-lncRNAs were imported to GREAT software (http://bejerano.stanford.edu/great/public/html/index.php) for co-location analysis. Neighboring coding genes were then matched with DE-mRNAs to obtain a co-expression dataset. Three subgroups of genes (DE-lncRNA co-located genes, DE-mRNAs, and co-expressed genes) were used for gene expression network analysis using Cytoscape software (v2.8.3).

## Supplementary Material

Supplementary Figure 1: Gene Ontology analysis of differentially expressed coding genes (DE-mRNAs).
Supplementary Table 1: List of differentially expressed long noncoding RNAs (lncRNAs) in Esophageal squamous cell carcinoma (ESCC).
Supplementary Table 2: List of differentially expressed coding RNAs in Esophageal squamous cell carcinoma (ESCC).

## Figures and Tables

**Table 1:** Validation of selected differential expression of mRNAs in esophageal squamous cell carcinoma in independent studies.

Probe name	*P* value	FC	Regulation	Gene symbol	Genbank accession	Independent study	Reference
A_33_P3232692	0.005838984	8.301461	Up	IL24	NM_001185156	Microarray	[20]
A_24_P411121	0.00055	5.329484	Up	TNFRSF18	NM_148901		
A_23_P169097	8.81*E* − 05	4.466178	Up	WISP1	NM_080838		
A_23_P304304	0.004822649	3.944957	Down	ARSF	NM_004042		
A_24_P56363	0.003573538	3.323955	Down	CAB39L	NM_030925		
A_23_P419760	0.001041661	32.70335	Down	CRISP3	NM_006061		
A_23_P413923	0.002921898	4.54899	Down	DMRTA1	NM_022160		
A_23_P56978	0.002093997	5.438183	Down	PTK6	NM_005975	RNA-seq	[21]
A_23_P115091	0.005171322	3.289834	Down	RAB25	NM_020387	Q-RT-PCR	[22]
A_33_P3258542	0.001039129	20.36035	Down	SPINK8	NM_001080525	Microarray	[20]

**Table 2:** List of identified co-located and co-expressed genes with differentially expressed lncRNAs in ESCC.

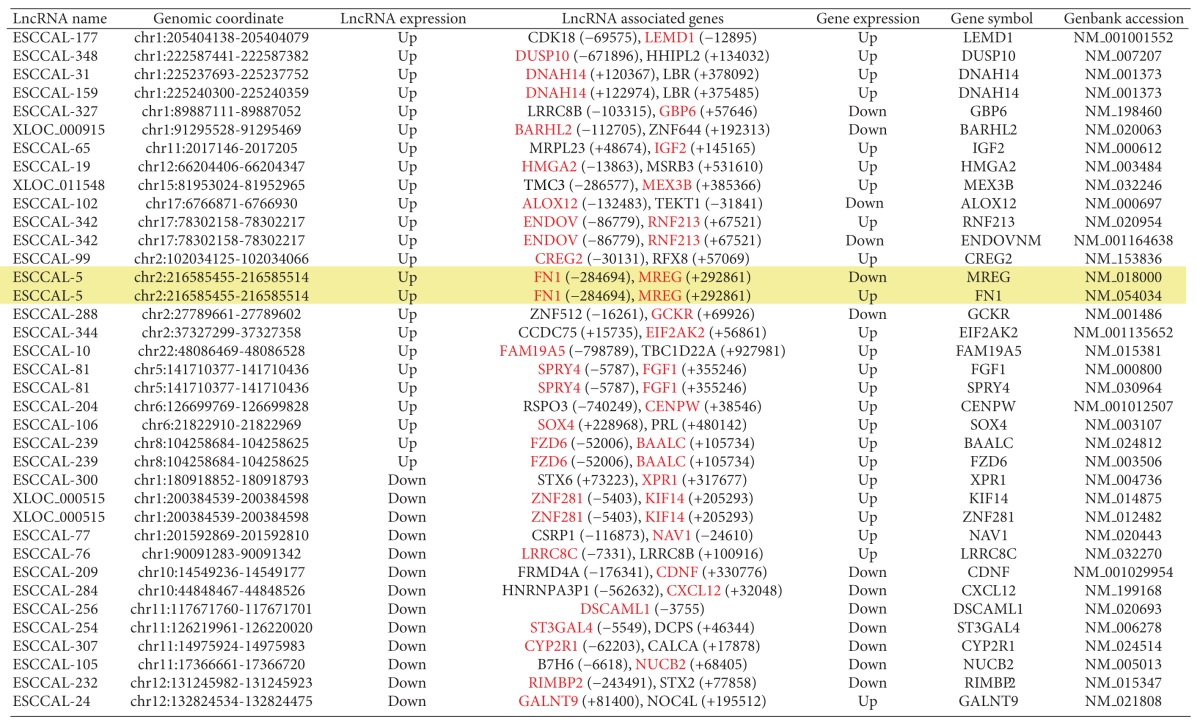 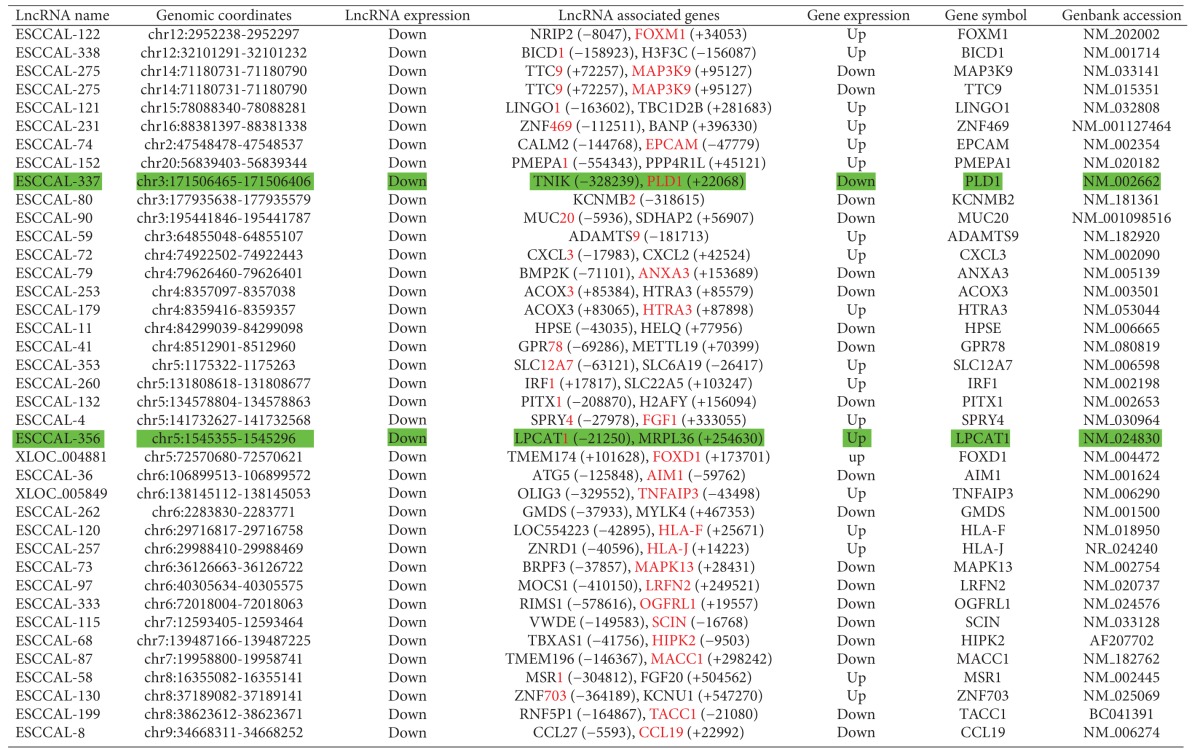

Notes: Red-highlighted genes are whose expressions are significantly changed in Esophageal Squamous Cell Carcinoma (ESCC). Green color-highlighted rows: genes involved in lipid metabolism predicted with GO enrichment analysis. Yellow color-highlighted rows: The expression of lncRNA ESCCAL-5 was validated by PCR.

## Abbreviations

AFAP1-AS1: Actin filament-associated protein 1 antisense RNA
ESCC: Esophageal squamous cell carcinoma
ESCCAL: ESCC-associated lncRNA
lncRNA: Long noncoding RNA
MALAT-1: Metastasis-associated lung adenocarcinoma transcript 1
HOTAIR: HOX antisense intergenic RNA
PCAT-1: Prostate cancer associated noncoding RNA transcript 1
PCR: Polymerase chain reaction.
