# Supplementary material for: Integrated Analysis of Long Noncoding RNA and Coding RNA Expression in Esophageal Squamous Cell Carcinoma
Source: Int J Genomics. 2013 Oct 7;2013:480534. doi: 10.1155/2013/480534 (PMC3814080; doi:10.1155/2013/480534)
Supplement: Supplementary file 1 — Supplementary Figure 1: Gene Ontology analysis of differentially expressed coding genes (DE-mRNAs). Supplementary Table 1: List of differentially expressed long noncoding RNAs (lncRNAs) in Esophageal squamous cell carcinoma (ESCC). Supplementary Table 2: List of differentially expressed coding RNAs in Esophageal squamous cell carcinoma (ESCC). [file IJG_Figure_S1_CFG_606395.pdf › IJG_Figure_S1_CFG_606395.pdf]

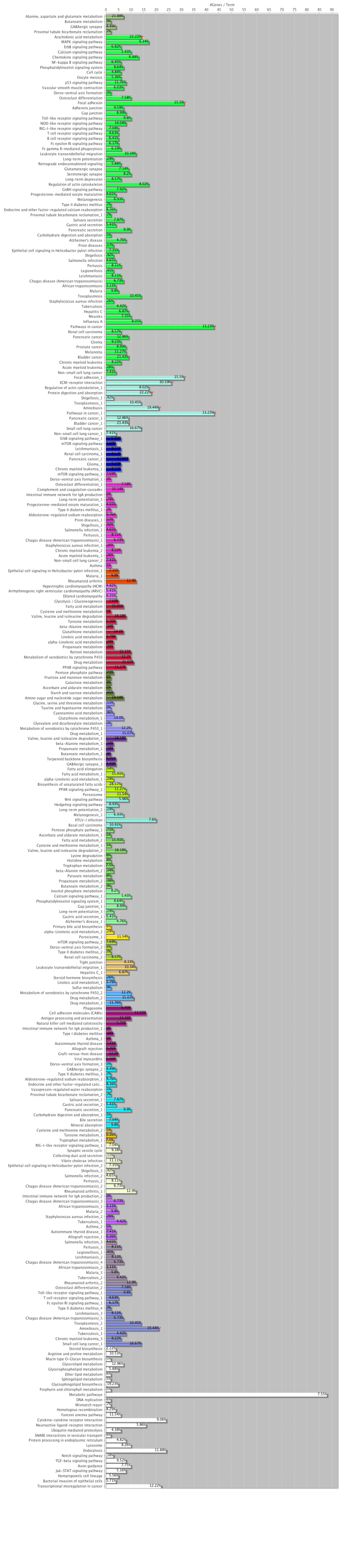
#Genes / Term
