# Supplementary material for: Integrated Analysis of Long Noncoding RNA and Coding RNA Expression in Esophageal Squamous Cell Carcinoma
Source: Int J Genomics. 2013 Oct 7;2013:480534. doi: 10.1155/2013/480534 (PMC3814080; doi:10.1155/2013/480534)
Supplement: Supplementary file 1 — Supplementary Figure 1: Gene Ontology analysis of differentially expressed coding genes (DE-mRNAs). Supplementary Table 1: List of differentially expressed long noncoding RNAs (lncRNAs) in Esophageal squamous cell carcinoma (ESCC). Supplementary Table 2: List of differentially expressed coding RNAs in Esophageal squamous cell carcinoma (ESCC). [file 480534.f1.zip › Supplementary_Info.pdf]

Supplementary Information

Integrated analysis of long non-coding RNA and coding RNA expression in esophageal squamous cell carcinoma

Wei Cao, Wei Wu, Fachun Shi, Xiaobing Chen, Lihua Wu, Ke Yang, Fu Tian, Minghui Zhu, Guoyong Chen, WeiWei Wang, Qinghe Xing, Fred G, Biddle, Jianqin Gu

Inventory

Figure S1, related to Figure 1D

Table S_DE-lncRNAs, related to Figure 1B

Table S_DE-mRNAs, related to Figure 1C

Supplementary Figure Legends

Supplementary Figure 1. Gene Ontology analysis of differentially expressed coding genes (DE-mRNAs). The list of 1219 DE-mRNAs was imported to ClueGo software for functional enrichment analysis. The functional terms with # genes/terms are listed as a chart. Statistically significant terms related to cancer are shown, such as arachidonic acid metabolism, focal adhesion, pathways in cancer, bladder cancer, focal adhesion-1, ECM-receptor interaction. The network was visualized with Cytoscape software and presented in text Figure 1D.

Supplementary Tables S1. List of differentially expressed long noncoding RNAs (lncRNAs) in Esophageal squamous cell carcinoma (ESCC). The changes in expression of lncRNAs between ESCC tissue and adjacent normal esophageal tissue was either greater or less than 2.0 fold and P value < 0.05 (P values were corrected for multiple testing using the method of Benjamini-Hochberg) were identified from microarray study. Most of the lncRNAs only have genomic coordinates (start and end position in the genome), these anonymous lncRNAs were named ESCC Associated Long noncoding RNAs (ESCCALs). Columns: the order of lncRNAs listed here are based on expression levels of fold changes (both upregulated or downregulated) from highest to lowest.

Supplementary Table S2. List of differentially expressed coding RNAs in Esophageal squamous cell carcinoma (ESCC). The changes in expression of coding RNAs between ESCC tissue and adjacent normal esophageal tissue was either greater or less than 2.0 fold and *P* value < 0.05 (*P* values were corrected for multiple testing using the method of Benjamini-Hochberg) were identified from microarray study. One mRNA gene may have multiple probes represented in the microarray.
